# The police hunch: the Bayesian brain, active inference, and the free energy principle in action

**DOI:** 10.3389/fpsyg.2024.1368265

**Published:** 2024-03-06

**Authors:** Gareth Stubbs, Karl Friston

**Affiliations:** ^1^Rabdan Academy, Abu Dhabi, United Arab Emirates; ^2^Institute of Neurology, University College London, London, United Kingdom

**Keywords:** active inference, Bayesian brain, intuition, policing, decision making, suspicion, free energy principle (FEP)

## Abstract

In the realm of law enforcement, the “police hunch” has long been a mysterious but crucial aspect of decision-making. Drawing on the developing framework of Active Inference from cognitive science, this theoretical article examines the genesis, mechanics, and implications of the police hunch. It argues that hunches – often vital in high-stakes situations – should not be described as mere intuitions, but as intricate products of our mind’s generative models. These models, shaped by observations of the social world and assimilated and enacted through active inference, seek to reduce surprise and make hunches an indispensable tool for officers, in exactly the same way that hypotheses are indispensable for scientists. However, the predictive validity of hunches is influenced by a range of factors, including experience and bias, thus warranting critical examination of their reliability. This article not only explores the formation of police hunches but also provides practical insights for officers and researchers on how to harness the power of active inference to fully understand policing decisions and subsequently explore new avenues for future research.

## Introduction

In what can be a high-stakes policing world, officers are often confronted with situations that demand swift yet informed decision-making. The ability to survive these complex and dynamic environments rests not only upon established operating procedures and training, but also on an officer’s intuition; their inherent capacity to generate “hunches”. These hunches, often perceived as *instinct* or *a gut feeling*, have long remained contested, even gaining the moniker: “suspicioning” (Quinton et al., 2000; Quinton, 2010, 2014; Hendy, 2021). Research in this area is related to stopping and searching, where the hunch directs a conscious choice to intervene with a driver or perform an outer clothing search for contraband or weapons (Alpert et al., 2005) and has often been collocated with race. Instead, this study focuses on the hunch as a more functional phenomenon that does not just arise at the point of discretionary choice but often as an involuntary response to sensory stimuli that cannot be readily explained.

Consider the following scenario.

*A police officer arrived at the scene of a recently reported domestic disturbance. The scene is chaotic, with a distraught woman seated on the curb and her bloodied head shedding clumps of hair because of the violent altercation that has just occurred. Her trembling voice informs the officer of a violent and angry male within the residence, while also emphatically warning that her two-year-old child remains trapped in the house. At this critical moment, the responding officer grapples with a decision that demands split-second judgment. As the officer approaches the door, goosebumps begin prickling the back of their neck, and an indescribable feeling emerges – a hunch that something is amiss. The door swings open, revealing a bare-chested and seemingly composed man who is well-built, in his thirties. He calmly invites the officer inside. Against a backdrop of uncertainty, the officer chooses to decline the invitation, and their internal alarm bells ring insistently.*

This scenario creates a fundamental question: How does an officer’s body generate these hunches, these internal alarm bells, and what role do they play in navigating the intricacies of the police experience? To explore this question, this article delves into the theoretical framework of Active Inference and the Free Energy Principle (FEP) (Parr et al., 2022) pioneered by the cognitive scientist Karl Friston et al. (2010). Active inference relies upon the brain operating as a Bayesian engine (Friston et al., 2011), continually generating and updating probabilistic models of the world, and actively intervening to minimize what is referred to as surprise. In this article, we examine how this concept is not only relevant but also indispensable for understanding the formation and implications of hunches in the context of policing. We dissect the generative processes within the officer’s brain, assisted by active inference, to reveal how hunches may serve as crucial instruments in their decision-making toolkits. This discussion then allows for the generation of a number of hypotheses.

This conceptual investigation not only sheds light on the cognitive mechanics of the police hunch but also offers practical insights into harnessing the power of active inference for the future policing research agenda.

### What is a hunch?

Taking the prior reference of suspicion as a starting point, we can begin to draw a more precise boundary around what could be called an officer’s *hunch*. Suspicion is a product of the officer’s interpretive view of the world, and although it may be based on prior experience, it is also based on a predicted generative model of what may happen in the future. An example, in simple terms, is:

**Police officer**: “*I want to stop that car; I do not like the look of it*.”

Such interactions with the public have been the subject of concentrated study (Alpert et al., 2005; Parks et al., 2005; Stroshine et al., 2008; Quinton, 2010, 2014), in which the discretion inherent in the decision becomes subject to scrutiny. Biases and stereotypes play a role in the formation of suspicion in these circumstances (Heussenstamm, 1971; Brown, 1981; Holmberg, 2000; Dunham et al., 2005; Stroshine et al., 2008), with aspects of the visual world influencing the formation of suspicion that leads to directed physical activity. Interestingly, there are also studies that illustrate suspicion as a moderator of correspondence bias (Fein, 1996), triggering active, sophisticated attribution rather than brute-force stereotyping.

This research evidence points only toward a small aspect of what could be considered a hunch, as the hunch does not just reside within discretionary stops and searches for individuals or vehicles. The hunch rests within an officer’s body and mind and is constantly available for deployment in any given circumstance. It can be present after or during a police interview, when compiling evidence at a crime scene, or upon arriving at the scene of a particularly violent domestic incident. It is a cognitive product of the interpretation of sensory input at any given moment, formed upon the figurative grounding of the officer’s gathered and interpreted experience.

These hunches are not restricted to police officers, and may manifest as the product of experiential “build-up” over time in many professions and encounters. Intuition has been studied within nursing (Buckingham and Adams, 2000), medicine more widely (Adler, 2022), firefighting (Okoli et al., 2016), and the armed forces (McCown, 2010). These studies discuss how specific circumstances within these professions may generate particular hunches, with little research available to cross tabulate these experiences across genres. If these hunches are caused by repeated exposure to incidents within those lines of work, it is fair to suggest that hunches themselves may be particular, and in some cases may even be harmful. An example of this may be when disproportionate experience may contribute toward the development of a disproportionate hunch (Hinton, 2017; Villiger, 2023).

If the police hunch can be considered wider than car stops and person searches, then the literature discussing its deployment is examining a small corner of what could be a much wider phenomenon. This has been discussed in the literature that defines suspicion as a cultural pillar (Bowling et al., 2019), a behavioral thread that runs through policing, rooted in the profession itself. The literature examining the operation of suspicion is international, and commonalities are present across international boundaries (Nickels and Verma, 2008). This may suggest a reliance upon a collectively built and reinforced *schema* (Smith et al., 2006) that rely upon scripts (Schank and Abelson, 2013). Schema and scripts are cognitive structures that represent organized knowledge about the world. They can involve events or processes that assist with guiding perception, interpretation, and action and represent a wider cognitive framework (the schema), and a more specific subtype (script). Scripts and schema enable individuals to anticipate courses of events in various situations, reducing cognitive load by providing a template for the user to access. This fits comfortably with an evolutionary psychology perspective and, in particular, cultural niche construction as the mechanism for co-constructing shareable world or generative models (e.g., Heyes, 2018; Constant et al., 2019; Vasil et al., 2020). These psychological phenomena describe a shared way of understanding and navigating the real world — sometimes referred to in terms of “common ground” and alignment of “frames of reference.” See for example (Allan, 2013; Fields et al., 2021; Friston et al., 2022).

This makes intuitive sense, as all police officers at some point will arrest people, stop cars, and enter violent situations. These experiences are not rare and make up a very normal day for the average uniform police officer in any corner of the world. However, they are not shared by other professions who do not experience physiological and cognitive reactions to entering such encounters. It is therefore not surprising that police officers who live through distinct experiences then form understandings of the world that are distinct from those that do not.

This gradual collection of distinct experiences then leads to a distinct development of cognitive processing, built upon experiences that non-officers do not share; to summarize, it theoretically sets officers cognitively apart from other professions. This was explored by Smith et al. (2006) in their investigation of whether prior experience influenced their application of suspicion, with gender providing some significant relationships with suspicion formation.

If this is the case, then it makes sense that any police officer experiencing a hunch is experiencing a cognitive event built upon the foundation of distinct experiences over time. To illustrate this, let us return to the initial scenario discussed at the start of this article.

*Due to the goosebumps on their neck, the police officer declines the bare-chested male’s invitation to come inside and instead takes hold of the closest arm, yanking him into the front garden. A violent encounter then ensues where the taser is deployed twice and the male is eventually subdued and arrested. Upon entering the property to check the welfare of the young child, his attention is drawn to the large kitchen knife that the male dropped as he was yanked into the garden. It was positioned in the porch directly behind the front door.*

This situation could be read as a complete chance, yet the officer will attest (they are the author of this article) that the feeling was quite different from anything they had felt before when attending hundreds of domestic incidents. Indeed, it was the sensory input that presented prior to the encounter which caused an embodied reaction construing, “*Something is wrong here*.” This then generated a behavior that may have looked hyper-aggressive to onlookers; there was little conversation before the male was forcibly dragged into the front garden, and a significant physical altercation took place. All this occurred as the direct product of a hunch, a feeling that the normal cognitive models of prediction were being subverted by *something* that could not be isolated or understood by the person experiencing them, yet immediately influenced behavior.

Returning to the other discussed example.

**Police officer**: “*I want to stop that car; I do not like the look of it*.”

This noticeably short descriptive statement describes the operationalization of a hunch by the officer in question. They describe that they wish to act to stop the car because they do not “like the look of it.” This is open to interpretive bias from an onlooker who does not have the foundational sensory input of a police officer, and it is possible to read this statement in a negative or even punitive manner. The officers’ choice of words leaves a great deal of descriptive information, which they themselves may not be able to sufficiently understand. It relies upon the same “*Something is wrong here*” feeling discussed in the scenario above, but how that feeling is generated is unobtainable to the officer experiencing them. This leads to a significant conundrum: How does the officer justify their hunch and what prior sensory input does their brain rely upon to influence their behaviors?

To answer this question, other literature was consulted in intuition, with the phenomenon described as follows:

“... *knowing without being able to explain how we know*” (Vaughan, 1979, p. 46).

This describes a cognitive output in the form of ‘knowing,’ with the person who receives it being subsequently unable to describe how they arrived at the intuition itself. In a literature review of intuition, Shirley and Langan-Fox (1996) described the following:

“…*individuals sometimes do not even realize that a process is taking place until a solution appears in consciousness. Most writers also agree that intuition is characterized by intense confidence in the intuitive feeling*.” (p. 596).

Intuition as a studied subject has garnered a great deal of interest and has been researched in many different settings (Svenson et al., 2023), from the business and management sector (Kopalle et al., 2023), into education (Suwarto et al., 2023), leadership (Hallo and Nguyen, 2021) and even machine learning (Dai et al., 2020). Interestingly, the hermeneutics of intuition in these research areas are linked with valuable tacit knowledge and experience, which are viewed positively. This contrasts with the attachment of suspicion as a referent lens in the policing research canon. It is possible that the balanced literature in other areas may illustrate some bias in the research, representing a divide between the police being viewed as oppressive enforcement/intervention (negative lens) and the police as a profession that may demand some aspects of operational creativity in decision making. As criminals continually innovate to stay ahead of the police (Aldridge and Décary-Hétu, 2014; Broadhurst, 2018), it is fair to suggest that police may have a fundamental requirement to be intuitive in following new avenues or approaches during their operations.

A meta-analysis examined the relationship between rational decision-making and intuition (Wang et al., 2017) constructs in psychology. This research indicates that they are separate constructs, and as such share few similarities, running upon dual cognitive systems, and often referred to as operating within the systems 1 and 2 thinking dichotomy (Stanovich et al., 2000). Intuitive decision making in this sense is described as “emotional” (system 1) rather than rational (system 2) thinking, with one of the main discerning features being the temporality of the decision making; how long as the decision maker got to make the decision within context. This has severe implications for policing, as much of the operational uniform activity is temporal in nature. How much time does an operational officer arriving at an incident have to make their operational decisions? The reliance on System 1 thinking in the police environment may be high, forcing an uneven emphasis on the development of high-speed, active decision-making.

Tentatively, with prior discussion in mind, the author posits a definition of a hunch for the following discussion:

*An often unbidden, salient intuition experienced by a police officer that subsequently influences their actions.*

This wording has been chosen purposely, as the hunch is often not consciously invited, only experienced. It is different to suspicion in the sense that it can arise in many different situations as a response to any kind of sensory stimulus, and the feeling within the officer is salient; it is prominently different to other “normal” or more rational day-to-day decision making. This distinction is important. If the brain is constantly generating hypotheses in response to stimuli, how does one distinguish between a hunch and any other hypothesis? The important word within the definition is “salience”: the officer recognizes (i.e., infers) something unique about a particular hypothesis. The nature of this difference is discussed later in this article, in relation to the imperatives for decision-making—that feature notions of salience and resolving uncertainty.

It is also clear that a hunch operates on a spectrum. On one hand, it is an unbidden feeling that subtly influences behavior, such as the feeling about a car that an officer wishes to stop. It is a minor feeling that on many occasions, is unexplainable and developed from previous experiences within that officer’s service. On the other hand, there is an immediate and severe feeling that requires far more active intervention in the environment, such as the domestic violence incident discussed; this is a hunch linked with personal survival.

To investigate the now defined hunch, it is important to consider how the brain cognitively generates these intuitions.

## Generating hunches: the Bayesian brain, active inference, and the free energy principle

### Bayesian reasoning

Bayesian reasoning is a mathematical approach that combines prior knowledge with incoming information, enabling the brain to develop and refine probabilistic models of the world continuously (Tiao and Zellner, 1964).^1^ This approach has been employed in various fields, including psychology and neuroscience, to analyze phenomena, such as motor skills and various mental processes (Bowers and Davis, 2012). The idea of Bayesian inference aligns with the notion that the brain probabilistically represents information, utilizing neural coding as a suitable metaphor for its operations (Brette, 2019). Bayesian models, as exemplified by Bayes’ theorem, allow the integration of existing beliefs with new evidence in a mathematically rigorous manner (Tiao and Zellner, 1964).

Bayesian reasoning is based on Bayes’ theorem, an equation that expresses the probabilistic connection between prior knowledge and observed data.

$P\left(H|E\right)=\frac{P\left(E|H\right)\cdot P\left(H\right)}{P\left(E\right)}$

In this context:

*P(H|E)* denotes the probability of the hypothesis given the evidence after observation.
*P(E|H)* represents the likelihood of encountering the evidence if the hypothesis is true.
*P(H)* represents the prior probability assigned to hypothesis.
*P(E)* represents the marginal likelihood of the observed evidence.

If we remain with Bayes only, and relate it to our prior example upon arrival at the reported domestic incident, this theorem would represent the following:

The police officer responds to a domestic disturbance where the incoming sensory stimuli mix with their prior experiences and contribute to an internal Bayesian computation in the brain. As the officer encounters a distraught woman and hears of a potentially violent individual within a residence, the brain begins to calculate the likelihood of future violence given the available evidence, denoted by *(P(E|H))*. Simultaneously, the officer’s prior knowledge and training contribute to developing the prior probability of a hunch *(P(H))*. The brain combines this information with the overall likelihood of observing the given evidence in any situation *(P(E))*. Using Bayes’ theorem, we can posit that the officer computes the posterior probability of the hunch being necessary, given sensory evidence *(P(H|E))*, and the sensory input is such that it results in a unique internal alert – an unbidden feeling that guides their subsequent actions and decisions.

Although this would offer us some insight into the officer’s decisions, it does assume to some extent that the brain is not acting in any way to actively affect the environment; it is represented as non-agentic and essentially a simple database that continually updates its probabilistic beliefs. To consider the brain functioning as an agent within an environment, it is necessary to consider how the Bayesian Theorem is made more ‘complete’ via the addition of Active Inference and the Free Energy Principle.

### Active inference and the free energy principle

Moving beyond traditional Bayesian reasoning, recent advancements in cognitive science have developed the concept of Active Inference (AI) as a corollary of the Free Energy Principle (FEP). This provides a richer and more complete model (Friston et al., 2016) to assist in understanding the generation and implications of hunches in police environments. AI extends the Bayesian brain concept by emphasizing not only the passive assimilation of sensory information but also the active engagement of the brain to generate and update probabilistic models of the world (Knill and Pouget, 2004). Referred to as a “Bayesian machine in action,” this framework views the brain as an entity that not only infers from sensory input but also actively intervenes to minimize “surprise,” aligning its internal model with the external environment (Knill and Pouget, 2004) via perception and aligning the external environment with its internal model (Friston et al., 2016), via action. There is some debate with regards to the nature of “action” and its epistemological (Rescorla, 2015) connotations. It involves the way that organisms coming to know and understand their world being inherently linked to their actions within it. Knowledge and understanding therefore, are not just related to passive observation, but are constructed through active engagement with the environment. This debate has implications for attempting to understand epistemology in biological systems; *knowing* can be seen an embodied, dialogic process.

The Free Energy Principle (FEP) is a foundational concept when considered alongside Active Inference and the Bayesian Brain framework. FEP suggests that living organisms, including the brain, strive to minimize the difference between their internal model of the world and the sensory inputs they receive (Friston, 2010), as scored by the extent to which the sensory inputs were surprising, given an internal (i.e., generative) model of what caused those inputs. AI, while building on traditional Bayesian principles, further accentuates this idea by highlighting that the brain not only processes information passively but also actively engages with the environment. It does this to minimize surprise, or what is essentially the mismatch between the brain’s predictions and actively sampled real-world observations (Friston et al., 2016). This unified framework emphasizes the brain’s continuous efforts to update its internal model, actively inferring and intervening to minimize free energy, which serves as a measure of surprise in ongoing interaction with the external world.

This surprise can be quantified mathematically in terms of the variational free energy (*F*), which is minimized during active inference (i.e., belief updating in the brain):


$$\begin{array}{c} Q(s,a)=\arg\min_{Q}F\\ F=E_{Q}[\ln\underset{posterior}{\underset{︸}{Q(s,a)}}-\ln\underset{likelihood}{\underset{︸}{P(o|s,a)}}-\ln\underset{prior}{\underset{︸}{P(s,a)}}]\\ =\underset{divergence}{\underset{︸}{D_{KL}[Q(s,a)||P(s,a|o)]}}-\ln\underset{evidence}{\underset{︸}{P(o)}}\\ =\underset{complexity}{\underset{︸}{D_{KL}[Q(s,a)||P(s,a)]}}-\underset{accuracy}{\underset{︸}{E_{Q}[\ln P(o|s,a)]}} \end{array}$$

In the context of this article, free energy provides a mathematical expression for surprise: when the brain is confronted with sensory observations (*o*) it updates its posterior belief Q(s,a) about the states of affairs causing those observations. Note that these states include the states of the world (*s*) and action upon that world (*a*). In the context of a police officer, these could be environmental factors and their behavior.

P(s,|a,|o) represents the true posterior probability based on sensory evidence to which the Bayesian beliefs converge (c.f., *P(H|E)* above – in this case the likelihood of future violence) under a model of how outcomes are caused. In the equation above, this generative model is expressed in terms of the likelihood of some observations P(o|,s|,a) , given their causes and prior beliefs P(s,a) about those causes (that include action). In short, Q(s,a) corresponds to the brain’s best guess about states of affairs that can be read as a hypothesis or hunch; namely, the best inference about what it expects to encounter in a given situation.

Technically, the above equation specifies Bayesian beliefs Q(s,a) – about states and actions – as those beliefs that minimize variational free energy, where variational free energy has been expressed in three equivalent forms; each affording a complementary interpretation. Because the (KL) divergences^2^ cannot be less than zero, the penultimate equality means that free energy is zero when the Bayesian belief is the true posterior. At this point, the free energy becomes the negative log evidence for the generative model (Beal, 2003). This means that minimizing free energy is equivalent to maximizing model evidence. Crucially, the negative log evidence is also known as surprisal or more simply surprise. Therefore, maximizing model evidence — also known as self-evidencing (Hohwy, 2016) — is equivalent to minimizing surprise by minimizing free energy.

Surprise quantifies the discrepancy between what the brain expects and what it observes. In the generation of a hunch, high surprise values indicate a significant deviation from the brain’s predictions, triggering an alert or heightened awareness. Conversely, lower surprise values suggest closer alignment between expectations and observations. This equation denotes that surprise is a value that can be modeled, allowing for possible prediction of hunch generation and severity. A hunch is not something that is ephemeral, it is the outcome of underlying Bayesian processing, and the generation of a world model that seek to actively infer and manage the presenting ‘level’ of surprise.

This is known as Bayesian belief updating, where prior beliefs about (unobservable) causes are updated into posterior beliefs after seeing (observable) consequences. For example, seeing a distressed woman outside her house (observable consequences) revises prior beliefs about domestic abuse (unobservable causes). With an updated belief about domestic abuse, seeing a victim in a state of distress is now less surprising.

This relative level of surprise can therefore be described as driving the brain to initiate active engagement with the outside world in order to drive changes in the observed environment. This causes an update to its internal model generation to align better with the now changed (and potentially more explainable) sensory evidence (Knill and Pouget, 2004; Constant et al., 2019). In this context, surprise is not merely a passive reaction to unexpected events but rather a driving force behind the brain’s continuous adjustment of predictive models based on real-time interactions and sensory stimuli. In the context of the policing hunch, AI suggests that an officer’s brain operates as a dynamic Bayesian engine and not merely as a passive recipient and processor of information (Friston et al., 2016). This active engagement allows for the continuous adjustment of officers’ predictive models based on real-time interactions and sensory stimuli. Technically, this means selecting courses of action that minimize the expected surprise following that action. Because expected surprise can be read as uncertainty, this means that action is compelled to resolve uncertainty, for example, making further inquiries to resolve uncertainty about whether domestic abuse is the right explanation for the evidence at hand. Please see Box 1 for a more technical explanation of active inference in terms of resolving risk and ambiguity.

BOX 1: **Active inference.**

Recent trends in theoretical neurobiology, machine learning, and artificial intelligence converge on a single imperative that explains both sense-making and decision-making in self-organizing systems, from cells (Friston et al., 2015) to cultures (Veissiere et al., 2019). This imperative is to maximize the evidence (a.k.a. marginal likelihood) for generative (a.k.a., world) models of how observations are caused. This imperative can be expressed as minimizing an evidence bound called variational free energy (Winn and Bishop, 2005) that comprises complexity and accuracy (Ramstead et al., 2022):

*Free energy = model complexity – model accuracy.*

Accuracy corresponds to goodness of fit, while complexity scores the divergence between prior beliefs (before seeing outcomes) and posterior beliefs (afterwards). In short, complexity scores the information gain or cost of changing one’s mind (in an information theoretic and thermodynamic sense, respectively). This means Bayesian belief updating is about finding an accurate explanation that is minimally complex (c.f., Occam’s principle). In an enactive setting—apt for explaining decision-making—beliefs about ‘which plan to commit to’ are based on the free energy expected under a plausible plan. This implicit planning as inference can be expressed as minimizing expected free energy (Friston et al., 2010):

*Expected free energy = risk (expected complexity) + ambiguity (expected inaccuracy).*

Risk is the divergence between probabilistic predictions about outcomes, given a plan, relative to prior preferences. Ambiguity is the expected inaccuracy. An alternative decomposition is especially interesting from the perspective of CNT:

*Expected free energy = expected cost* – *expected information gain.*

The expected information gain underwrites the principles of optimal Bayesian design (Lindley, 1956; Fields et al., 2021), while expected cost underwrites Bayesian decision theory (Berger, 2011; Fields et al., 2021; Gklezakos and Rao, 2022). However, there is a twist that distinguishes active inference from expected utility theory. In active inference, there is no single, privileged outcome that furnishes a utility or cost function. Rather, utilities are replaced by preferences, quantified by the (log) likelihood of encountering *every* aspect of an observable outcomes. In short, active inference appeals to two kinds of Bayes optimality and subsumes information and preference-seeking behavior under a single objective.

To summarize the previous discussion,

- The officer’s collected sensory input was predicted using the officer’s generative model.
- They do not align properly, this denotes surprise
- The severity of surprise generates a hunch that something is wrong
- The FEP at work motivates action – through AI – that seeks to resolve uncertainty
- Surprise is minimized bringing predictions back into alignment with the sensory input

This process ends with the building of the experience-based generative model in the police officer prior to any further incident attended. According to Bayes’ theorem, this indicates that prior experience moderates the boundary or magnitude of surprises experienced by police officers. In short, reliance upon experience within particular specialisms in policing may be far more important than previously thought for decision-making, long-term police capability, and well-being.

Note that exactly the same principles operate in our example over different timescales and levels of analysis. One can read the officer’s further investigation as being motivated to resolve uncertainty to best explain the evidence at hand. This is an example of active inference at a deliberative, propositional level that could be entered into an incident report. However, the same mechanisms also operate at a sub-personal level of gut feeling, which may be less easy to describe. For example, the fast, emotional (i.e., type) inference “*that something is wrong*” is a perfectly appropriate hypothesis to both cause and explain the surprising sensation of prickling goosebumps. The very notion of “gut feelings” speaks to active inference — not about extra personal states of affairs — but about the officers’ own body. This is known as interoceptive inference, and may be a key component of hunches, in which the officer uses their body as an additional source of evidence that the situation is surprising. This raises the notion that not only will the Bayesian brain be receiving external stimuli through which to denote surprise, it is also receiving embodied stimuli (Holzer, 2017). Both of these stimuli will manifest in different ways, in different situations, calling for an active resolution of ambiguity and minimization of risk of an interoceptive and exteroceptive sort. Interoceptive inference—along these lines—has many interesting aspects (Seth, 2013; Gu and FitzGerald, 2014; Barrett and Simmons, 2015). At its simplest, one can read the minimization of surprise as homeostasis and the commitment to pre-emptive plans—that elude the need for homeostatic responses—as allostasis (Pezzulo et al., 2015; Barrett et al., 2016; Seth and Tsakiris, 2018). Indeed, many suppose that an interoceptive component of active sensing is necessary to lend an emotional (i.e., valence) aspect to belief updating (Seth and Friston, 2016; Fotopoulou and Tsakiris, 2017; Duquette, 2020). Box 1 provides a definition of ambiguity and risk, and see Barrett and Simmons (2015), Gu and FitzGerald (2014), Seth (2013) and Seth and Friston (2016) for an introduction to interoceptive inference.

Note further that in active inference, both volitional and autonomic responses are the causes and consequences of sensory input. In other words, active inference rests upon a circular causality implicit in making sense of observations that are generated through behavior. For example, the volitional act of approaching the door causes it to “*swing open*.” Similarly, autonomic responses to the feeling that “*something is wrong*” produces goosebumps that provides further evidence that “*something is wrong*.”

### Hunches and niche construction

In light of this discussion, how this may manifest over longer periods is relevant. The Bayesian Brain, FEP and AI have all been discussed within the area of niche construction (Constant et al., 2018), where organisms experience long-term influence over their immediate environment (their niche) and are able to enter into what could be considered “low surprise,” longitudinal relationships with their sensory inputs around them. This assists with the building of sustaining generative models in their brain that provide a reliable service for their organism, both controlling and altering the environment, while always taking in and adapting to the modified, but mostly predictable, sensory inputs. Unfortunately, the nature of police work does a great deal to prevent this function from being properly developed in the officer, which may result in the particular development of the hunch as a more sensitive alarm system than in other professions. This problem is discussed in the following section.

Within the dynamic environment of law enforcement, officers routinely confront swiftly unfolding, unique situations. The luxury of formulating protracted causal generative models is a rarity, as every attendance to a call for police is, to some extent, novel. The concept of cognitive niche construction is directly relevant to this peculiarity, as it discusses the process whereby organisms – herein, police officers – articulate and sustain cause-effect models (Constant et al., 2018) of their immediate environment as guiding frameworks for their behavior. In contrast to scenarios in ecological niche construction, where organisms may adapt to their environment over very long periods of time, officers grapple with very constrained temporal parameters. The requirement to make instantaneous decisions and navigate these novel, often risky, circumstances creates a necessity for highly temporal niche construction. This requires rapid modification of the selective and novel environment during police encounters.

This temporal facet of niche construction is particularly relevant for understanding the cognitive intricacies underpinning hunch generation within policing. Officers’ capacity to construct and promptly update cause-effect models of their immediate environment emerges as a vital adaptive mechanism. Police officers attending an incident often have no knowledge of the people, environment, or circumstances behind the call. Each call therefore represents a potential risk to their safety, and it is only through repeated attendance at calls over time that their predictive modeling becomes sufficiently general to accept more novel, higher bounds of surprise within their volatile work environment. The ability to adapt quickly and actively manipulate sensory input is fundamental to a police officer, and this phenomenon is present within the cultural literature, often discussed as pragmatism:

“*Pragmatism, with its emphasis on getting things done and a focus on outcomes rather than processes, can create a culture that values action over reflection*.” (Loftus, 2010, p. 305).

Despite what can be quite a negative lens in cultural literature, if the theories discussed prior are in operation, this is a very likely outcome within police culture. Officers use their brains daily to adapt to incidents with high surprisal bounds (see Box 1), forcing their brains to generate action that is far more ‘active’ than in many other lines of work, especially in responding to epistemic affordances in a risk-sensitive fashion. It is possible to hypothesize that this characteristic has indeed been ‘trained’ into officers’ brains over time, and in turn bled out into their organizational practice and decision making. Other studies discuss this as a reliance upon command within police decision-making, when other methods of leadership may be more appropriate (Grint, 2010).

In general police work therefore, police officers do not have the luxury of longer-term, active involvement in their work environment. There are likely to be high levels of surprise and unpredictability, as the calls they are responding to will often not contain actors with which police officers are familiar. This will leave a requirement for the officers to rely heavily on prior beliefs and encourage far more active involvement within the niche environments they attend, thus gaining an immediate return on surprise minimization. The hunch is as a result more highly “tuned” than in other areas of work; officers need it to assist with the minimization of free energy (the gaining of immediate control), whilst surrounded by higher than normal levels of surprise.

**Figure 1:**
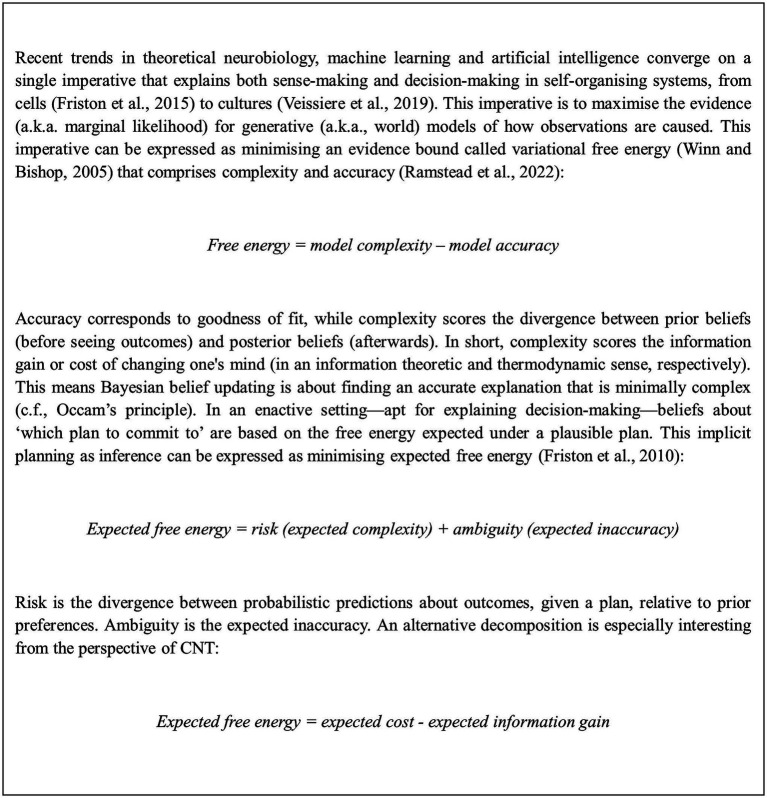
Hypothesis.

This discussion generates the following hypotheses (Figure 1).

## Discussion

If the theories of the Bayesian Brain, FEP and AI are integrated into the police context, cognitive science and neuroscience can be combined to explain what the brain is doing when hunches arise, why they arise, and how the agent in the world (the police officer) then becomes active in modeling their immediate environment. This is a more general approach for describing what the process of policing *does.* Although it is possible to describe police in terms of crime reduction, crime response, crime detection, and the maintenance of public order, the need to understand policing at a cognitive level can sometimes be missed in contemporary research.

To illustrate this, the continuing example of the domestic incident is explored. It is likely that this incident will be reduced to a crime statistic for the purposes of most research, or at the most, an anecdote in interview-based research, yet the experience of the most powerful hunch an officer ever felt is left uninvestigated. Several questions can be posed.

- What cognitive signals were generated by initial attendance that contributed to the hunch generation?
- When the Bayesian brain processes these signals, what is produced empirically during the process of active inference? What was the officer’s qualia?
- How were these qualia interpreted – there is likely to be a hermeneutic of hunches among active police officers? How can this be examined and shared?
- How did the officer justify this hunch in later court proceedings? How did this influence the writing of police statements and other court documents?

This list is non-exhaustive but shows the potential for solid research in this area. Perhaps more importantly, it delves into what can be considered the *art* of policing. The consideration of these variables is referred to as combinatorically explosive because of the endlessly developing pot of variables leaking into the Bayesian brain (Kleiter, 1992). The police brain is remarkably able to recognize and distil signals received at a rapid pace and interpret them into actions that minimize incredibly elevated levels of surprise. This is worthy of extensive study.

At an even wider level, it may be possible to push the theories of AI and FEP into calls for police services. It is possible that when the public calls for police, their ability to actively navigate and influence their immediate environment has reached its limit. The police become third-party interventions that deliver a return to homeostasis for the caller, which acts as a tool for the caller to minimize their free energy and bring their lives back into the bounds of predictability. Strangely, this returns to earlier research by scholars such as Bittner, who describes policing as dealing with the following:

“*…something that ought not to be happening, and about which someone had better do something now*,” Bittner (1990, p. 24).

Although not within the direct scope of the preceding discussion, this quote encapsulates AI and FEP acting at a societal level. This has been discussed in other literature as working within ‘Regimes of Expectation’ (Constant et al., 2019), where predictability is constructed at a much wider scale than the individual. These can be described as shared expectations of what can be construed as normal – the police act as agents of returning the world to a certain level of normality.

From the micro-cognitive functions of the hunch to the much wider use of the hunch as a potential tool for officers to navigate surprise on behalf of the members of the public who call for their service, the ground is set for a series of thematic investigations.

### Harnessing the hunch for future police research

To conclude this article, we return to the example of the domestic incident.

The police officer’s response to the reported domestic disturbance can be evaluated using the frameworks of the Bayesian Brain, the Free Energy Principle (FEP), and Active Inference (AI). The Bayesian Brain continuously models probabilities, taking into account the prior likelihood of potential threats as the officer arrives at the chaotic scene. The FEP comes into play as the officer’s brain strives to minimize surprise, as evinced by volitional action (e.g., making further inquiries) and autonomic responses (e.g., goose bumps). This surprise triggers an adaptive response guided by AI, as the officer actively decides not to enter a location that they would usually go inside. This demonstrates the ability of the brain to dynamically adjust predictive models in response to real-time environmental stimuli. Therefore, the hunch played a crucial role in shaping officers’ split-second decisions in dealing with complex and unpredictable situations. It is unclear what experiences the officer had during the incident provided their brain with anomalous data, heightening their awareness of potential risk, and encouraging the immediate use of active inference to minimize surprise.

From the prior investigation, the exact nature of the experience of hunches in policing is key to this study. The point where a particular hypothesis becomes salient is rooted in subjective experience, referred to as *qualia* (Shoemaker, 1990). It is possible that hunches in particular circumstances are raised into the experiencer’s consciousness in very particular ways, and this area of study could be highly beneficial to police officers in many different settings. This is a contested area of scholarship because of the oft-referred ‘hard problem’ of consciousness itself (Chalmers, 2017), making it a highly fruitful area for further investigation. In active inference, qualia are generally read as the qualitative experience that attends recognizing a change in perceptual or attentional set, which is accompanied by instantiating top-down (inductive or empirical) priors that necessarily change the precision of lower belief updating. The associated phenomenology therefore rests on a kind of mental or covert action. Please see (Limanowski and Blankenburg, 2013; Limanowski and Friston, 2018; Sandved-Smith et al., 2021), for discussion.

To summarize, it is reasonable to assert that this procedure occurs on a spectrum at every police attendance. Officers must confront unfamiliar circumstances to establish some degree of control over the environment. This topic has been explored extensively in various police research contexts. However, without incorporating the previously mentioned concepts, it remains unclear what the brain, and therefore the police officer, is attempting to accomplish cognitively. To identify and investigate potential avenues for further research in this area, the following ten topics listed in Table 1 are proposed.

**Table 1:** Areas of future research.

1) Longitudinal Study on Hunch Development:
This would involve a longitudinal study tracking the development of hunches in police officers as their career develops. It would investigate how cumulative experiences over time contribute to the refinement and reliability of hunches.
2) Comparative Analysis with Other Professions:
This research would compare the prevalence and effectiveness of hunches in police officers with professionals in other dynamic environments (e.g., the business world where hunches have been construed positively as intuition). This could help with exploring whether the unique demands of police work lead to distinctive hunch patterns.
3) Neurobiological Correlates of Hunches:
It is possible to collaborate with neuroscientists to explore the neurobiological processes associated with hunch formation. This would require the use of brain and body imaging techniques to identify specific regions activated during hunch-related decision-making.
4) The Impact of Training on Hunch Accuracy:
Investigating the role of training programs in shaping and enhancing the accuracy of hunches would illuminate whether specific training interventions can improve officers’ ability to generate reliable hunches.
5) Diversity and Bias in Hunches:
This research would explore the impact of diversity (in terms of gender, race, etc.) on the formation and reliability of hunches. As an example, this would provide some insight into potential gendered hunches and their implications for policing practices.
6) Operationalization of Hunches in Policing:
This research would examine how hunches are operationalized in real-time police decision-making. Using both qualitative and quantitative methods, it could investigate the factors that contribute to the activation and follow-through of hunches during specific incidents.
7) Training for Surprise Management:
Using action research, it is possible to develop and evaluate training programs aimed at improving officers’ ability to manage and respond to surprise effectively. This could assist with assessing how surprise management training impacts the generation and utilization of hunches in good police work, and exploring how surprise is emotionally regulated in officers in active deployment.
8) Comparative Analysis of Policing Models:
This research would compare the prevalence and effectiveness of hunches in different policing models (community policing, predictive policing, etc.), investigating how organizational structures and obligations influence hunch development and utilization.
9) Impact of Technological Support:
This research could explore how emerging technologies, such as predictive analytics and artificial intelligence, influence the generation and reliance on hunches in policing. Assess the potential benefits and challenges associated with integrating technology into hunch-based decision-making.
10) Public Perception and Trust:
Finally, it is possible to investigate how public perception and trust in law enforcement are influenced by officers’ reliance on hunches, and the communication of how they are being used during investigations or incidents. It would examine the role of transparency and communication in shaping public attitudes toward hunch-based decisions.

## Data availability statement

The original contributions presented in the study are included in the article/supplementary material, further inquiries can be directed to the corresponding author.

## Author contributions

GS: Writing – original draft, Writing – review & editing. KF: Conceptualization, Investigation, Supervision, Visualization, Writing – review & editing.

## Footnotes

^1^Reasoning as a term will be explained within this section and has not been used prior in this article as it is peculiar to Bayes Theorem – yet to be properly explored.

^2^The KL divergence can be used to quantify the “surprise” or “free energy” of a system, given its model of the world. In this context, the brain aims to minimize this divergence (technical) measure of surprise in order to reduce the difference between the expected (internal model) and actual (sensory inputs) states. Minimising this divergence (free energy) measure of surprise (e.g., prediction error) is thought to underpin both perception (updating the internal model to better match sensory inputs) and any action (modifying the environment to better match the internal model).
